# Implementation of robotic rectal cancer surgery: a cross-sectional nationwide study

**DOI:** 10.1007/s00464-022-09568-1

**Published:** 2022-08-30

**Authors:** L. J. X. Giesen, J. W. T. Dekker, M. Verseveld, R. M. P. H. Crolla, G. P. van der Schelling, C. Verhoef, P. B. Olthof

**Affiliations:** 1 Department of Surgery, Erasmus MC Cancer Institute, Rotterdam, The Netherlands; 2 Department of Surgery, Reinier de Graaf Gasthuis, Delft, The Netherlands; 3 Department of Surgery, Franciscus Gasthuis & Vlietland, Rotterdam, The Netherlands; 4 Department of Surgery, Amphia Hospital, Breda, The Netherlands

## Abstract

**Aim:**

An increasing number of centers have implemented a robotic surgical program for rectal cancer. Several randomized controls trials have shown similar oncological and postoperative outcomes compared to standard laparoscopic resections. While introducing a robot rectal resection program seems safe, there are no data regarding implementation on a nationwide scale. Since 2018 robot resections are separately registered in the mandatory Dutch Colorectal Audit. The present study aims to evaluate the trend in the implementation of robotic resections (RR) for rectal cancer relative to laparoscopic rectal resections (LRR) in the Netherlands between 2018 and 2020 and to compare the differences in outcomes between the operative approaches.

**Methods:**

Patients with rectal cancer who underwent surgical resection between 2018 and 2020 were selected from the Dutch Colorectal Audit. The data included patient characteristics, disease characteristics, surgical procedure details, postoperative outcomes. The outcomes included any complication within 90 days after surgery; data were categorized according to surgical approach.

**Results:**

Between 2018 and 2020, 6330 patients were included in the analyses. 1146 patients underwent a RR (18%), 3312 patients a LRR (51%), 526 (8%) an open rectal resection, 641 a TaTME (10%), and 705 had a local resection (11%). The proportion of males and distal tumors was higher in the RR compared to the LRR. Over time, the proportion of robotic procedures increased from 15% (95% confidence intervals (CI) 13–16%) in 2018 to 22% (95% CI 20–24%) in 2020. Conversion rate was lower in the robotic group [4% (95% CI 3–5%) versus 7% (95% CI 6–8%)]. Anastomotic leakage rate was similar with 16%. Defunctioning ileostomies were more common in the RR group [42% (95% CI 38–46%) versus 29% (95% CI 26–31%)].

**Conclusion:**

Rectal resections are increasingly being performed through a robot-assisted approach in the Netherlands. The proportion of males and low rectal cancers was higher in RR compared to LRR. Overall outcomes were comparable, while conversion rate was lower in RR, the proportion of defunctioning ileostomies was higher compared to LRR.

**Supplementary Information:**

The online version contains supplementary material available at 10.1007/s00464-022-09568-1.

Over the past decades the treatment of rectal cancer has shifted toward more minimally invasive treatment strategies. One of the innovations was the introduction of laparoscopic rectal cancer surgery. Several randomized trials showed at least similar oncological outcomes when compared to open surgery and with a faster short-term recovery [1, 2]. Earlier, the implementation of laparoscopic surgery for rectal cancer was opposed because of a longer operative time and uncertain oncological outcomes. These issues are not debated anymore [3–5].

More recently, similar concerns have been raised with the implementation of robot-assisted rectal cancer surgery [6, 7]. The articulating instruments and the stable 3D camera image using a robotic system facilitate procedures in the narrow space of the pelvis. Therefore, the robotic approach may have advantages over the laparoscopic approach with non-articulating straight instruments. Also, robotic surgery seems to be ergonomically superior to laparoscopic surgery [8]. The first randomized trial to compare the robotic with the laparoscopic approach could not show a benefit in conversion rate and a later trial showed similar outcomes for the completeness of the total mesorectal specimen [9]. Later meta-analyses of the available studies showed similar short-term and oncological outcomes, but did show a lower conversion rate in robotic procedures [1, 9–11].

In the Netherlands, an increasing number of centers have implemented a robotic surgical program for rectal cancer [12]. A recent report shows that despite a learning curve, implementation of a robotic rectal resection program is safe and shows even possible favorable outcomes compared to standard laparoscopy [13]. The present study aims to evaluate the trend in the implementation of robotic resections (RR) for rectal cancer relative to laparoscopic rectal resections (LRR) in the Netherlands between 2018 and 2020 and to compare the differences in outcomes between the operative approaches.

## Methods

All patients diagnosed with rectal cancer who underwent a rectal resection between 2018 and 2020 in the Netherlands were included. Data were extracted from the mandatory Dutch ColoRectal Cancer audit (DRCA). The DCRA is a nationwide audit that collects information on key patient-, diagnostic-, and operative characteristics in combination with postoperative outcomes that allows continuous feedback and analyses of performance indicators. Data entry is web based in a highly secured database with the majority of colorectal surgeons recording the data themselves. Further details regarding data collection in the DCRA and methodology have been published before [14, 15]. The information is based on evidence-based guidelines and is cross-checked with data from the Netherlands Cancer Registry. All data are anonymized and no informed consent was needed. Ethical approval was not needed according to Dutch Law. The study proposal was approved by the scientific committee of the DCRA.

In the Netherlands, robotic surgery is usually implemented in a stepwise approach. First, surgeons completed the intuitive training program. After this surgeons are proctored for the the initial cases without a predefined number of procedures. In 2020, there were 35 Davinci surgical systems in the Netherlands across 27 hospitals. The number of centers performing RR were not available due to data anonymization.

The data extracted from the DCRA database included patient characteristics, disease characteristics, surgical procedure details, and postoperative outcomes. The outcomes included any complication within 90 days after surgery, readmission within 90 days after surgery, and mortality within 90 days after surgery. Anastomotic leakage was defined as leakage or abscess formation at the level of the anastomosis as observed on imaging, endoscopy or during surgery. Reintervention was defined as every invasive reintervention (surgical, endoscopic, or radiological) for the treatment of a complication and consequently defined as Dindo grade III.

Data were categorized according to registered surgical approach (robotic, laparoscopic, open, transanal total mesorectal excision (TaTME) or local surgery) and the year of surgery. Patients who underwent a low anterior resection were categorized in patients with a partial mesorectal excision (PME) or total mesorectal excision (TME). All missing values were 10% or less and no imputation was conducted.

Categorical data were presented as numbers with percentages in tables and proportions with 95% confidence intervals in text, which were calculated according to the Wald interval method. Differences in categorical variables were tested using chi-square or Fisher’s exact tests. Continuous data were tested for normality using the Shapiro–Wilk test. All continuous data were presented as median with inter-quartile-range (IQR) and differences were tested using, the Mann–Whitney *U* test or Kruskal–Wallis test were used. Uni- and multivariable analysis for were performed using logistic regression analysis. All relevant confounding variables from the univariable analysis were included in the multivariable analysis. All statistical analyses were performed using SPSS (Version 26.0, IBM, Chicago, IL).

## Results

In total, 6533 patients underwent a resection for rectal cancer in the study period. No clear/definite surgical approach could be retrieved for 203 patients (3%) and these patients were excluded. The remaining 6330 patients were included in the analyses.

Between 2018 and 2020, 1146 patients underwent a RR (18%), 3312 patients LRR (51%), and 526 (8%) an open rectal resection. The remaining patient underwent either taTME (641 patients, 10%) or a local resection (705 patients, 11%). Patients and disease characteristics as well as outcomes stratified by treatment approach are shown in Table 1. Compared to the patients with LRR, there were more male patients in the RR group [65% (62–67) versus 61% (59–62)] and the proportion of distal tumors < 5 cm from the anal verge was higher [44% (41–47) versus 41% (39–42)]. Conversion rate was lower in the robotic group [4% (3–5) versus 7% (6–8)]. Conversion was termed strategic in 72% (60–85) in RR, and 66% (60–72) after LRR (34/47 versus 162/244, *P* = 0.499) and the other conversion were reactive. For patients who underwent TME, the proportion of patients who received an anastomosis was similar in the robotic and laparoscopic group (77% (74–80) versus 75% (73–77), Table 2). Defunctioning ileostomies were more common in the robotic group [42% (38–46) versus 29% (26–31)]. Anastomotic leakage rate was similar in both groups [16% (12–19) versus 16% (14–18)].

**Table 1:** Characteristics and outcomes of patients who underwent rectal resection

	Robot *N* = 1146	Laparoscopy *N* = 3312	*P* value	Open *N* = 526	Local *N* = 705	TaTME *N *= 641
Age, median (IQR)	67 (59–75)	68 (59–75)	0.035	66 (56–74)	69 (61–75)	65 (27–73)
Male sex, *n* (%)	742 (65)	2009 (61)	0.015	310 (59)	459 (65)	447 (70)
ASA ≥ III, *n* (%)	266 (23)	807 (24)	0.431	137 (26)	194 (28)	135 (21)
cT, *n* (%)			0.273			
1	41 (4)	123 (4)		6 (1)	323 (46)	30 (5)
2	280 (24)	847 (26)		32 (6)	236 (34)	163 (25)
3	708 (62)	1970 (59)		240 (46)	79 (11)	400 (62)
4	98 (9)	282 (9)		232 (44)	5 (1)	37 (6)
x	19 (2)	90 (3)		16 (3)	62 (9)	11 (2)
cN, *n* (%)			0.003			
0	560 (49)	1682 (51)		145 (28)	609 (86)	314 (49)
1	344 (30)	979 (30)		159 (30)	36 (5)	189 (29)
2	239 (21)	604 (18)		211 (40)	13 (2)	134 (21)
x	3 (0)	47 (1)		11 (2)	47 (6)	4 (1)
cM1, *n* (%)	75 (7)	181 (5)	0.176	102 (19)	6 (1)	43 (7)
Tumor height, *n* (%)			< 0.001			
< 5 cm	507 (44)	1341 (41)		274 (52)	357 (51)	367 (57)
5–10 cm	391 (34)	1074 (32)		134 (25)	176 (25)	192 (30)
Neoadjuvant, *n* (%)			0.504			
5 × 5 Gy	314 (27)	821 (25)		108 (21)	32 (5)	176 (28)
Chemoradiotherapy	348 (30)	1031 (31)		321 (61)	49 (7)	193 (30)
Presurgery stoma, *n* (%)			< 0.001			
Defunctioning colostomy	76 (7)	126 (4)		121 (23)	–	8 (1)
Defunctioning ileostomy	5 (0)	21 (1)		13 (3)	1 (0)	4 (1)
Elective procedure, *n* (%)	1113 (97)	3264 (99)	0.002	466 (89)	704 (100)	637 (99)
Procedure, *n* (%)			< 0.001			
PME	120 (10)	616 (19)		42 (8)	–	40 (6)
TME	679 (59)	1652 (50)		205 (39)	–	680 (85)
APR	339 (30)	994 (30)		252 (28)	–	339 (30)
Other	8 (1)	50 (2)		27 (5)	–	16 (2)
Conversion, *n* (%)	47 (4)	244 (7)	0.003	–	–	–
pT, *n* (%)			< 0.001			
0	68 (6)	204 (6)		43 (8)	76 (11)	56 (9)
1–3	1024 (89)	2997 (90)		375 (71)	607 (88)	578 (90)
4	32 (3)	104 (3)		108 (21)	1 (0)	7 (1)
X	22 (2)	7 (0)		–	21 (3)	–
pN, *N* (%)			0.267			
0	697 (61)	2155 (65)		306 (58)	428 (61)	420 (7)
1	332 (29)	887 (27)		161 (31)	28 (4)	171 (27)
2	67 (6)	225 (7)		56 (11)	4 (1)	46 (7)
X	50 (4)	45 (1)		3 (1)	245 (35)	4 (1)
Number of nodes, *n* (%)	15 (12–22)	15 (12–22)	0.682	15 (11–21)	–	16 (12–22)
Positive margin, *n* (%)	16 (1)	68 (2)	0.189	31 (6)	22 (3)	6 (1)
90-Day morbidity, *n* (%)	483 (42)	1133 (34)	< 0.001	312 (59)	104 (15)	297 (46)
Reintervention rate, *n* (%)	196 (17)	515 (16)	0.216	113 (21)	46 (7)	135 (21)
Hospital stay, days, median (IQR)	6 (4–9)	5 (4–8)	< 0.001	9 (6–15)	1 (1–2)	6 (4–10)
90-Day readmission, *n* (%)	195 (17)	507 (15)	0.312	107 (20)	74 (10)	147 (23)
90-Day mortality, *n* (%)	11 (1)	37 (1)	0.778	9 (2)	4 (1)	6 (1)

**Table 2:** Characteristics and outcomes of patients who underwent robotic rectal resection in 2018, 2019, and 2020 in the Netherlands

	2018 *N *= 340	2019 *N *= 372	2020 *N *= 434	*P* value
Age, median (IQR)	67 (59–74)	68 (59–76)	67 (57–75)	0.478
Male sex, *n* (%)	223 (66)	231 (62)	288 (66)	0.418
ASA ≥ III, *n* (%)	70 (21)	86 (23)	110 (25)	0.298
cT, *n* (%)				0.389
1 2 3 4 x	9 (3) 89 (26) 212 (62) 27 (8) 3 (1)	11 (3) 88 (24) 239 (64) 28 (8) 6 (2)	21 (5) 103 (24) 257 (59) 43 (10) 10 (2)	
cN, *n* (%)				0.731
0 1 2 x	162 (48) 109 (32) 67 (20) 2 (1)	182 (49) 109 (29) 81 (22) –	216 (50) 126 (29) 91 (21) 1 (0)	
cM1, *n* (%)	20 (6)	22 (6)	33 (8)	0.253
Tumor height, *n* (%)				0.010
< 5 cm 5–10 cm	148 (44) 126 (37)	161 (43) 128 (34)	198 (47) 137 (32)	
Neoadjuvant, *n* (%)				0.260
5 × 5 Gy Chemoradiotherapy	98 (29) 109 (32)	90 (24) 120 (32)	126 (29) 119 (27)	
Procedure, *n* (%)				0.265
PME TME APR Other	27 (8) 217 (64) 93 (27) 3 (1)	45 (12) 204 (55) 121 (33) 2 (1)	48 (11) 258 (59) 125 (29) 3 (1)	
Conversion, *n* (%)	21 (6)	12 (3)	13 (3)	0.525
pT, *n* (%)				< 0.001
0 1–3 4 X	30 (9) 288 (85) 7 (2) 15 (4)	19 (5) 341 (92) 9 (2) 3 (1)	19 (4) 395 (91) 16 (4) 4 (1)	
pN, *n* (%)				< 0.001
0 1 2 X	187 (55) 94 (28) 18 (5) 41 (12)	236 (63) 111 (30) 21 (6) 4 (1)	274 (63) 127 (29) 28 (6) 5 (1)	
Number of nodes, *n* (%)	15 (11–22)	16 (12–22)	16 (12–23)	0.575
Positive margin, *n* (%)	5 (1)	3 (1)	8 (2)	0.453
90-Day morbidity, *n* (%)	146 (43)	152 (41)	185 (43)	0.826
Reintervention rate, *n* (%)	52 (15)	67 (18)	77 (18)	0.570
Hospital stay, days, median (IQR	6 (4–10)	6 (4–9)	5 (4–8)	0.005
90-Day readmission, *n* (%)	58 (17)	72 (19)	65 (15)	0.243
90-Day mortality, *n* (%)	4 (1)	2 (1)	5 (1)	0.596

Over time, the proportion of robotic procedures increased from 15 (13–16) in 2018 to 22% (20–24) in 2020 (Table 3). On the other hand the rate of TaTME and local resection decreased from 12 (11–14) to 9% (7–10) and from 12 (11–13) to 9% (8–11), respectively. The proportion of laparoscopic (51–52%) and open (8–9%) procedures remained stable for the study period. The rate of neoadjuvant chemo radiotherapy remained stable over time.

**Table 3:** Anastomosis, defunctioning ileostomy, and anastomotic leakage rates in patients who underwent total or partial mesorectal excision according to the surgical approach

	Robot	Laparoscopy	*P* value	Open	TaTME
TME	679	1652		205	547
Anastomosis	522 (77)	1243 (75)	0.403	119 (58)	466 (85)
Defunctioning ileostomy	220 (42)	359 (29)	< 0.001	32 (27)	248 (53)
Anastomotic leakage	81 (16)	196 (16)	0.895	17 (14)	87 (19)
PME	120	616		42	40
Anastomosis	109 (91)	500 (81)	0.010	18 (43)	20 (50)
Defunctioning ileostomy	25 (23)	96 (19)	0.376	1 (6)	8 (40)
Anastomotic leakage	10 (9)	48 (10)	0.891	4 (22)	3 (15)

When comparing the robotic procedures per year, most patient and disease characteristics remained similar (Table 2). The proportion of patients who underwent robotic rectal resection for low tumors increased. The outcomes remained stable over time, the number of retrieved lymph nodes, margin status, morbidity, readmissions, and mortality did not change over the studied years. Hospital stay did drop with 1 day from a median 6 (4–10) in 2018 to 5 (4–8) days in 2020 (*P* = 0.005).

In patients with low tumors < 5 cm from the anal verge who underwent a low anterior resection, the proportion of patients with an anastomosis was 61% (55–68) in the robotic and 64% (61–69) laparoscopic group. Defunctioning ileostomies were created in 58% (50–67) of the robotic cases and 33% (28–38) of the laparoscopic cases with an anastomosis. The anastomotic leak rate was 23% (16–30) in the robotic group and 21% (16–25) in the laparoscopic group.

For patients with a tumor between 5 and 10 cm who underwent a low anterior resection, the anastomosis rate was 83% (79–87) in the robotic and 78% (76–81) in the laparoscopic group). Defunctioning ileostomies were created in 41% (36–47) of the robotic and 29% (25–32) of the laparoscopic cases with an anastomosis and the leakage rate was 14% (10–18) in the robotic and 15% (13–18) in the laparoscopic group (*P* = 0.637).

For patients with a tumor above 10 cm who underwent a low anterior resection, the anastomosis rate was similar in the robotic [91% (87–95)] and laparoscopic group [87% (84–90)]. Defunctioning ileostomy and anastomotic leak rates were 21% (15–28) versus 17% (14–21), and 9% (5–14) versus 6% (4–8).

After correction for relevant confounders, a robotic approach was not associated with an increase in the creation of an anastomosis compared to a laparoscopic approach (Table S2). When correcting for confounders in patients with an anastomosis, a robotic approach was not associated with a difference in anastomotic leakage rate compared to the laparoscopic approach (Table S3).

Over the three studied years in the robotic cases a lower percentage of anastomoses were created, the use of defunctioning ileostomies declined drastically from 50 (43–56) to 28% (22–34), and the anastomotic leakage rate remained stable (Table 4). For the laparoscopic resections the anastomosis rate and anastomotic leakage rate was similar over the study period. The use of defunctioning ileostomies was less frequent with laparoscopy and while the use declined over time, de decrease was lower compared to the robotic group.

**Table 4:** Anastomosis, defunctioning ileostomy, and anastomotic leakage rates in patients who underwent robotic and laparoscopic rectal resection in 2018, 2019, and 2020 excluding APR and total colectomies

	2018	2019	2020	*P* value
Robotic				
Anastomosis	203 (83)	204 (82)	225 (74)	0.011
Defunctioning ileostomy	102 (50)	84 (41)	64 (28)	< 0.001
Anastomotic leakage	28 (14)	27 (13)	39 (17)	0.429
Laparoscopic				
Anastomosis	662 (77)	582 (76)	513 (77)	0.961
Defunctioning ileostomy	196 (30)	154 (26)	110 (21)	0.006
Anastomotic leakage	93 (14)	74 (13)	77 (15)	0.542

The 90-day morbidity rate was higher after RR compared to LRR [42% (39–45) versus 34% (33–36)] while reintervention rate was similar [17% (15–19) versus 16% (14–17)]. Specific complication were shown in Table 5. At multivariable analysis RR remained associated with increased morbidity over LRR (Table s4), while the rates were similar for reintervention rate (Table s5).

**Table 5:** Complications after rectal resection according to approach

	Robot *N *= 1146	Laparoscopy *N *= 3312	*P* value	Open *N *= 526	Local *N *= 705	TaTME *N *= 641
Non-anastomotic abscess,* n* (%)	85 (7)	169 (5)	0.004	60 (11)	8 (1)	46 (7)
Bleeding,* n* (%)	16 (1)	46 (1)	0.986	11 (2)	29 (4)	9 (1)
Ileus,* n* (%)	108 (9)	220 (7)	0.002	52 (10)	15 (2)	54 (8)
Fascial dehiscence,* n* (%)	13 (1)	21 (1)	0.093	16 (3)	1 (0)	2 (0)
Bowel perforation,* n* (%)	6 (1)	15 (0)	0.763	6 (1)	9 (1)	2 (0)
Urinary leakage,* n* (%)	9 (1)	21 (1)	0.589	13 (2)	1 (0)	2 (0)
Surgical site infection,* n* (%)	73 (6)	160 (5)	0.044	75 (14)	9 (1)	21 (3)
Pulmonary complication,* n* (%)	38 (3)	95 (3)	0.443	46 (9)	2 (0)	27 (4)
Cardiac complication,* n* (%)	32 (3)	81 (2)	0.520	26 (5)	3 (0)	12 (2)
Thromboembolic complication,* n* (%)	10 (1)	24 (1)	0.620	9 (2)	1 (0)	4 (1)
Infectious complication*,* n* (%)	66 (6)	162 (5)	0.250	56 (11)	9 (1)	31 (5)
Neurologic complication,* n* (%)	16 (1)	30 (1)	0.157	16 (3)	1 (0)	7 (1)
Other complication,* n* (%)	173 (15)	340 (10)	< 0.001	85 (16)	24 (3)	96 (15)

*Any infectious complication other than pulmonary or surgery related

## Discussion

The present cohort shows an increase in robotic rectal resections in the Netherlands between 2018 and 2020. The proportion of males and low tumors was slightly higher in the robotic surgery group. Otherwise, the RR group was similar to the LRR group. The proportion of patients with an anastomosis and the rate of anastomotic leakage was similar between the RR and LRR groups. Robotic surgery was associated with less frequent conversions. Positive margins and number of retrieved nodes were similar, while overall complications were more prevalent in the RR group.

TME surgery has become the gold standard for rectal cancer, this technique was implemented in the era of open surgery [16, 17]. Since then, the development and advances in laparoscopic surgery have led to the introduction of laparoscopy for more demanding procedures including rectal resection [18]. While there were initial concerns on the oncological outcomes of LRR, subsequent larger randomized trials that have compared laparoscopic to open rectal cancer resection demonstrated similar positive margin, overall survival and local recurrence rates [19–21]. As expected, LRR is associated with faster recovery compared to open surgery [5]. Furthermore LRR seems beneficial compared to open especially in frail older patients due to a reduction in cardiopulmonary complications [22].

LRR is technically challenging, the confined space of the low pelvis allows limited working space. The unstable hand held camera, straight instruments, and poor ergonomic position for the surgeon all in part cause a considerable learning curve [23, 24]. The robotic approach might overcome these limitations, with a stable 3D camera image, instruments with endo-wrists, elimination of tremor and an ergonomic position of the surgeon [25, 26]. These theoretical advantages are balanced by a longer operative time, higher costs, and again a new learning curve [27, 28]. In the Netherlands, an increasing number of centers have implemented a robotic surgical program for rectal cancer [12]. Most reports show comparable short-term and oncological outcomes compared to laparoscopic surgery [29–32]. TaTME is another technique that tries to overcome the previously mentioned limitations of LRR, especially in low rectal cancer. While this technique has been a topic of debate due to high local recurrence rates that were reported by a study from Norway, recent studies show similar oncological outcomes in expert centers compared to LRR and RR [33–35]. Our data show that, in contrast to RR, TaTME does not spread throughout the Netherlands.

In the present cohort, there were little differences in the patient and disease characteristics between the RR and LRR group. However, the proportion of distal tumors and males was slightly higher in the RR group.

This could suggest that centers prefer to perform a RR when the resection is expected to be more technically challenging [13, 36]. Unfortunately, since a hospital variable was not available, the distribution of approaches across centers could not be assessed.

The proportion of robotic rectal cancer procedures increased from 15 in 2018 to 22% in 2020 and the absolute number of robotic procedures increased from 340 in 2018 to 434 in 2020. Although these rates are in line with a nationwide Korean analysis and a larger United States cohort, there is no rapid widespread implementation of robotic rectal cancer surgery [36–38]. The total rates of minimal invasive procedures in this cohort are higher compared to other nationwide series. While 92% of all procedures were performed minimally invasive, these rates were 27% in Switzerland, 40% in Germany, 63% in the USA, 80% in Korea [36, 39]. The relatively slow introduction of robotic procedure might be explained by the associated costs compared to LRR. [27]. As a result the majority of the hospitals in the Netherlands have not yet acquired a surgical robot Moreover, up to now most outcomes of RR are not superior to LRR with similar long-term and short-term outcomes [29, 31, 32, 40, 41].

Yet, the conversion rate has been reported to be lower in robotic rectal resection compared to laparoscopy with rates of 2–7% compared to 8–16% [10, 11]. These rates are similar to the 4 versus 7% reported in this unselected cohort. While this was statistically significant the difference is less than reported in literature, this might be due to the high minimal invasive surgery rate in this cohort. As reported before the rate of minimally invasive colorectal surgery in the Netherlands is close to 90% and a decrease in conversion rate at a national level is observed over time [42]. Furthermore, the numbers in our cohort suggest robotic rectal surgery was implemented in more hospitals over the last years. When implementing a new complex technique, it is likely that the learning curve has some influence on peri- and postoperative outcomes. A recent study has demonstrated lower conversion rates for robotic rectal resections in a Dutch cohort with surgeons with extensive robotic experience [43].

No difference was found in anastomotic leakage rates between RR and LRR, this is in concordance with previous studies [9, 32]. A lower tumor distance from the anal verge has been identified as an independent risk factor for anastomotic leakage [44, 45]. This is also observed in the present cohort, anastomotic leakage was more common in patients with lower rectal cancers, from 23% in patients with a tumor < 5 cm from the anal verge to 9% for tumors > 10 cm from the anal verge. At multivariable analysis, the anastomotic leakage rate was similar for RR and LLR.

Patients who underwent TME with a RR were not more likely to have an anastomosis than LRR patients. In literature, there are few reports on the tendency to create an anastomosis in robotic and laparoscopic rectal resection. A recent report shows a higher rate of primary anastomosis in RR compared to LRR in robotic expert centers, but this could not be confirmed in this paper including a multivariable analysis, which was not performed in the recent study [33]. Defunctioning stomas were more common in the robotic group, compared to the laparoscopic approach. This is likely indicative of caution that comes with the implementation of a new technique since the use of ileostomies decreased rapidly over time to rates comparable to the laparoscopic approach in the latest robotic procedures.

A higher morbidity rate was observed in the RR compared to that with LRR, while reintervention rate was similar and these observations were upheld at multivariable analyses. The higher rate of minor complications after RR is likely due to higher ileus and SSI rates, as well as complications specified as other. Additional data on these complications was not available and the overall differences were small and might not be clinically relevant. Prospective studies should evaluate the true impact of RR on morbidity compared to LRR. This study has several limitations which are mainly due to the retrospective study design that is subject to selection. In addition, a center variable was not available as an item in the database due to privacy reasons. Consequently, the distribution of surgical approaches across centers could not be analyzed. Before 2018, there was no variable that categorized robotic procedures apart from conventional laparoscopic, which is why no procedures could be included before 2018. Furthermore, this report only provides short-term outcomes of rectal cancer surgery in the Netherlands. The strength of this study is the nationwide cohort and real time data, that includes all rectal resections performed in the Netherlands over a 3 year period.

In conclusion, rectal resections are increasingly being performed through a robot-assisted approach in the Netherlands. Besides more low tumors and more males in the robotic group, no clear patient selection was identified between the robotic and laparoscopic approach. Overall outcomes were comparable, while conversion rate was lower in the robotic group, overall morbidity was higher and hospital stay longer in the robotic group. Future studies including the center-specific use of robotic rectal resection could provide more insight into the implementation of robotic surgery. A comparison of the outcomes of RR and LRR should be approached with caution here, considering both the vast experience and the high rate of LRR in the Netherlands, and the learning curve and implementation of RR**.** So far, the uptake of robotic rectal resection in the Netherlands seems to be safe.

## Supplementary Information

Below is the link to the electronic supplementary material.

Supplementary file1 (DOCX 30 KB)
